# Evaluation of Modulators of cAMP-Response in Terms of Their Impact on Cell Cycle and Mitochondrial Activity of *Leishmania donovani*

**DOI:** 10.3389/fphar.2020.00782

**Published:** 2020-05-29

**Authors:** Amrita Saha, Anindita Bhattacharjee, Amit Vij, Pijush K. Das, Arijit Bhattacharya, Arunima Biswas

**Affiliations:** ^1^Infectious Diseases and Immunology, CSIR-Indian Institute of Chemical Biology, Kolkata, India; ^2^Department of Zoology, Cell and Molecular Biology Laboratory, University of Kalyani, Kalyani, India; ^3^Department of Microbiology, School of Life Sciences and Biotechnology, Adamas University, Kolkata, India

**Keywords:** *Leishmania*, cAMP, cyclic nucleotide phosphodiesterase, cell cycle, motility, etazolate

## Abstract

With the identification of novel cAMP binding effector molecules in *Trypanosoma*, the role of cAMP in kinetoplastida parasites gained an intriguing breakthrough. Despite earlier demonstrations of the role of cAMP in the survival of *Leishmania* during macrophage infection, there is essential need to specifically clarify the involvement of cAMP in various cellular processes in the parasite. In this context, we sought to gain a comprehensive understanding of the effect of cAMP analogs and cAMP-cyclic nucleotide phosphodiesterase (PDE) inhibitors on proliferation of log phase parasites. Administration of both hydrolyzable (8-pCPT-cAMP) and nonhydrolyzable analogs (Sp-8-pCPT-cAMPS) of cAMP resulted in a significant decrease of *Leishmania* proliferation. Among the various PDE inhibitors, etazolate was found to be potently antiproliferative. BrdU cell proliferation and K/N/F-enumeration microscopic study revealed that both cAMP analogs and selective PDE inhibitors resulted in significant cell cycle arrest at G_1_ phase with reduced S-phase population. Furthermore, careful examination of the ﬂagellar motility patterns revealed significantly reduced coordinated forward flagellar movement of the promastigotes with a concomitant decrease in cellular ATP levels. Alongside, 8-pCPT-cAMP and PDE inhibitors etazolate and trequinsin showed marked reduction in mitochondrial membrane potential. Treatment of etazolate at subcytotoxic concentration to infected macrophages significantly reduced parasite burden, and administration of etazolate to *Leishmania*-infected BALB/c mice showed reduced liver and spleen parasite burden. Collectively, these results imply involvement of cAMP in various crucial processes paving the avenue for developing potent antileishmanial agent.

## Introduction

Leishmaniasis, defined as an array of diseases including self-healing cutaneous leishmaniasis and fatal visceral leishmaniasis, is caused by various species of protozoan parasite *Leishmania*. Till date, the disease is considered as a neglected tropical disease and urgently requires new therapeutic interventions. For leishmaniasis, pentavalent antimonials have remained the standard medication till recent decades, but drug resistance is becoming an increasing problem in the disease prone areas (Croft et al., 2006). Although a number of new compounds, such as miltefosine or amphothericin B, have been developed over the last few years (Chappuis et al., 2007; Croft, 2008; Santos et al., 2008; Goto and Lindoso, 2010; Richard and Werbovetz, 2010), effective, safe, and cost-efficient chemotherapy of leishmaniases still remains elusive. It has been currently postulated that cAMP plays an important role in various cellular processes linked with survival and virulence of kinetopalstida parasites (Salmon et al., 2012; Tagoe et al., 2015). In *Trypanosoma brucei* detailed studies revealed that hydrolyzed products of cAMP as well as various isoenzymes of cyclic nucleotide phosphodiesterases (PDE) and adenylyl cyclase (AC) have an important role in parasite survival, virulence, and cell–cell communications (Salmon et al., 2012; Tagoe et al., 2015). Moreover, the isoform PDEC from *T. cruzi* was shown to be involved in osmoregulation, and its potential to be a drug target was validated by high throughput screening of a series of PDE inhibitors (Wang et al., 2012), which led to the identification of tetrahydrophthalazinone compound A (Cpd A) (Salmon et al., 2012). These observations underpin the possibilities of cAMP pathway as a candidate target for drug development (Wang et al., 2012).

*Leishmania* differs in terms of its repertoire of cAMP-pathway components from *T. brucei* as the number of AC genes is considerably less. Additionally it encodes one cytosolic heme-containing AC which is absent in *Trypansoma*, indicating the presence of subcellular micro-domain specific cAMP signaling in this parasite (Sanchez et al., 1995; Sen Santara et al., 2013). Elevation of cAMP was found to be a prerequisite for differentiation condition induced cell cycle arrest (G1) and adaptation against oxidative stress encountered during early macrophage invasion (Bhattacharya et al., 2008; Biswas et al., 2017). Moreover, the crucial roles played by both exogenous and endogenous cAMP in chemotaxis by sensing chemical cues and inducing flagellar wave reversal (Mukhopadhyay and Dey, 2016) suggest the existence of definitive cAMP regulated mechanisms in the parasite. Similar to other kinetoplastida parasites, *Leishmania* encodes at least seven different cyclic nucleotide targeted phosphodiesterases (PDE). X-ray crystallography structure of *L. major* PDEB1 and B2 has been determined, and it’s superposition with human PDEs revealed the presence of a unique subpocket nearby inhibitor binding sites (Wang et al., 2007), widening the scope of selective drug designing. The differentially regulated *L. donovani* PDEA was characterized to be a high K_M_ cytosolic PDE that regulates cytosolic cAMP pool and modulate expression of antioxidant genes (Bhattacharya et al., 2009). The other cytosolic PDE, PDED, was found to be a moderate K_M_ enzyme that interacts with PKA-catalytic subunit like proteins to regulate cAMP level (Vij et al., 2014). Early studies showed that three human PDE inhibitors (etazolate, dipyridamole, and trequinsin) inhibit the proliferation of *L. major* promastigotes and *L. infantum*amastigotes with IC_50_ values in the range of 30–100 µM (Johner et al., 2006). Cytosolic isoform of leishmanial phosphodiesterase A (LdPDEA) showed a K_M_ of 166.6 µM and a Vmax of 0.98 µmol/mg/min with cAMP as substrate, and inhibitors like etazolate showed potent inhibition towards LdPDEA while trequinsin and dipyridamole showed moderate inhibition (Bhattacharya et al., 2009). On the other hand, kinetic analysis of the bacterially expressed LdPDED revealed a K_M_ of 25.89 μM and a Vmax of 5.2 µmol/mg/min when cAMP was used as a substrate. A number of important human PDE inhibitors like IBMX, EHNA, etazolate, zaprinast, and others as described by Vij et al., 2014 were tested against recombinant LdPDED of which rolipram showed moderate inhibitory activity (IC_50_ for LdPDED 21.6 ± 1.9 µM) (Vij et al., 2014). A robust PDE inhibitor library has been reported emphasizing PDE as exploitable targets for intervention (Sebastián-Pérez et al., 2018). However, a comprehensive profile for the impact of PDE inhibitors on various cellular processes in *Leishmania* is still lacking (Bhattacharya et al., 2009).

Although several cAMP and cAMP pathway associated phenotypes have been identified in *Leishmania*, any functional effector for cAMP is yet to be identified in this parasite. With an intention to assess the potential of cAMP pathway as target for chemotherapeutic intervention, in the present study, we intended to screen various cAMP analogs and PDE inhibitors. It was observed that hydrolysis-resistant cell-permeable cAMP analogs possess potent antiproliferative effect and show G_0_/G_1_ phase arrest indicating that unlike in *Trypanosoma*, where products of cAMP hydrolysis seemed vital for parasite transformation, in *Leishmania*, cAMP itself could induce these effects. Moreover, mammalian PDE4 inhibitor rolipram and etazolate and PDE5 inhibitor dipyridamole showed more antiproliferative effect and cell cycle arrest compared to the other well-known mammalian PDE inhibitors. Such effects were also found to be associated with slow and sluggish movement of *Leishmania* promastigotes, a change in ATP level and a striking change in mitochondrial membrane potential, reinforcing the need for further exploration of cAMP pathway in the parasite for drug development. Efficacy of etazolate in a mouse model of visceral leishmaniasis (VL) and apparent unresponsiveness of mammalian cells against this PDE inhibitor validate etazolate as a prospective repurposed drug against *Leishmania*.

## Materials and Methods

### Ethics Statement

The entire study was in strict accordance with the recommendations in the Guide for the Care and Use of Laboratory Animals of the Committee for the Purpose of Control and Supervision of Experiments on Animals (CPCSEA). The protocol was approved by the Institutional Animal Ethics Committee on Animal Experiments of the University of Kalyani (892/GO/Re/S/01/CPCSEA).

### Parasite Culture and Infection

The pathogenic strain of *Leishmania donovani* AG83 (MHOM/IN/1993/Ag83) was maintained in BALB/c mice, and promastigotes were cultured in medium 199 (M199; Invitrogen, Carlsbad, CA, USA) with Hank’s salt which contains Hepes buffer (12 mM), L-glutamine (20 mM), 10% heat-inactivated fetal calf serum, 50 U/ml penicillin and 50 µg/ml streptomycin. The promastigotes were isolated from infected spleens of BALB/c mice by culturing the respective spleens in M199 at 22°C for 5 days. Simultaneously, RAW 264.7, an adherent murine macrophage cell line was cultured in RPMI 1640 (Invitrogen) supplemented with 10% fetal calf serum (FCS), 100 U/ml penicillin, and 100 µg/ml streptomycin at 37°C with 5% CO_2_. For *in vivo* infection, female BALB/c mice of approximately 20 g of body weight were injected with 1 × 10^7^ stationary phase promastigotes of *L. donovani via* the tail vein. Etazolate at a dose of 10 mg/kg/day was administered daily over a period of 30 days following 2 weeks of post-infection. Parasites were isolated by aseptically removing the spleen and liver of the infected mice, and parasite burdens were assessed by Leishman–Donovan units (LDUs) by calculating the number of parasites per 1,000 nucleated cells × organ weight (g).

Inhibitors like etazolate, rolipram, trequinsin were soluble both in ethanol and dimethyl sulfoxide (DMSO). To maintain uniformity, all the PDE inhibitors were dissolved in DMSO, but the dimethyl sulfoxide concentration in the final assay solutions never exceeded 2%.

### Parasite Viability Assay

Parasite viability was measured by incubating the treated and control cells in 0.5 mg/ml 3-(4,5-dimethylthiazol-2-yl)-2, 5-diphenyltetrazolium bromide (MTT) for 3 h followed by subsequent addition of 100 µl of 0.04 N HCl in isopropyl alcohol. The principle underlying the MTT assay is that living mitochondria convert MTT to a dark blue compound, formazan, which is soluble in acid isopropyl alcohol. Formazan was detected at 570 nm on the microplate reader. The percentage viability was obtained by calculating the ratio of OD values in wells with treated cells versus the wells with controls multiplied by 100 (Miller et al., 2000).

### Cell Motility

Live *Leishmania* cells were visualized under bright field of Olympus IX 81 microscope with FV1000 confocal system on grooved slides which provides the freedom of movement to the parasites. Flagellar movements of both treated and control cells were analyzed (Singh et al., 2014). Numerous fields were used, and the distribution of counting areas was chosen in a nonbiased manner along the total surface area of the cover-slip for graphical representation of the motile parasites.

### Flow Cytometry and Cell Cycle Analysis

5 ml of parasite (0.5–1 × 10^7^ cells/ml) was centrifuged at 1,000 g for 10 min at 22°C, washed twice in PBS, and re-suspended in 70% ice-cold methanol for the purpose of fixation, and the cells were stored at −20°C for future use. The cells were treated with 20 mg/ml of RNase A and incubated at 37°C for 1 h before proceeding to analysis. Followed by this, propidium iodide was added for staining the DNA, and 20,000 cells were analyzed for DNA content using BD FACS Aria III, and the distribution of G_1_, S, and G_2_/M phases was then calculated from each histogram in BD FACS Diva Software.

### Macrophage Infectivity Assay

RAW 264.7 cells were cultured in RPMI-1640 and 10% FCS, counted, centrifuged at 1,500 rpm for 10 min at 4°C and resuspended in culture medium and distributed over 18 mm^2^ coverslips and cultured overnight. Cells were treated with PDE inhibitors as mentioned in the *RESULT* section and infected with *L. donovani* promastigotes at a ratio of ten parasites per macrophage, and the infection was allowed to proceed overnight. For the determination of the parasite number within the macrophage, cells were fixed in methanol and then stained with DAPI (4′,6-diamidino-2-phenylindole) (1 µg/ml) in PBS containing 10 µg/ml of RNase A (Sigma-Aldrich). Cells were visualized using a Olympus IX 81 microscope with FV1000 confocal system using a 100× oil immersion Plan Apo (N.A. 1.45) objective and analyzed by Olympus Fluoview Software (version 3.1a, Tokyo, Japan).

### BrdU Incorporation Assay and 1N/1K/1F Enumeration Assay

Promastigotes were treated with different concentrations of cAMP analogs and PDE inhibitors followed by 2 h incubation with 100 µM of 5-bromo-2-Deoxyuridine (BrdU) as per standard protocol, and the percentage BrdU incorporation was studied by BrdU cell proliferation assay (Millipore). For the determination of the number and position of nucleus (N), kinetoplast (K), and flagellum (F) in both treated and nontreated cells, parasites were harvested by centrifugation at 1,000 g for 10 min at 4°C, washed with 1× PBS, and fixed on a slide with methanol followed by DAPI (4′,6-diamidino-2-phenylindole) (1 µg/ml) in PBS containing 10 µg/ml of RNase A (Sigma-Aldrich) to stain nuclear and kinetoplast DNA and analyzed immediately. Images were acquired using an Olympus BX61 microscope at 1,000× magnification and an Olympus DP71 digital camera and were analyzed using Image Pro Plus Software (Media Cybrernetics).

### ATP Bio-Luminescent Assay and Measurement of Mitochondrial Membrane Potential

Parasites were centrifuged at 1,000 g for 10 min at 22°C, resuspended in medium and treated with cAMP analogs and PDE inhibitors, and the levels of cellular ATP production were studied by ATP bio-luminescent assay (Cayman Chemicals) as per the kit’s protocol.

Mitochondrial membrane potential was checked by JC-1 FACS analysis (BD Mitoscreen Mitochondrial Membrane Potential Detection Kit, BD Biosciences). Treated and nontreated cells were incubated with JC-1, a mitochondrial vital dye, for 15–20 min, washed with PBS, and prepared for flow cytometry. Cells were studied in BD FACS Canto, and BD FACS Diva software was used for studying the dot plots.

### Statistical Analysis

All the results shown are representatives of a number of independent experiments as mentioned in the figure legends. Statistical differences among the sets of data were determined by Student’s t-test with a P-value <0.05 considered to be significant.

## Results

### Effects of cAMP Analogs and PDE Inhibitors on L. donovani Promastigotes

The effect of various cAMP analogs and PDE inhibitors on the growth of log-phase promastigotes of *L. donovani* was measured by proliferation assays where parasites were grown in the presence of various concentrations of these compounds. Various methods implicated in determining drug susceptibility for *Leishmania* promastigotes involve monitoring growth by direct OD_600_ measurement (Bhattacharya et al., 2019) or by a more sensitive measurement using constitutively reporter protein expression strains like mcherry expressing promastigotes (Vacchina and Morales, 2014). Alternatively redox indicator like alamer blue (Mikus and Steverding, 2000) or MTT (Antwi et al., 2019) is applied, reduction of which depends on metabolic activity. Often there are differences between the levels of responsiveness determined with these methods. In this context, we determined the EC_50_ value with MTT (EC_50__MTT_) where 24 h of PDE-inhibitor exposures were followed by MTT assay and also by measuring OD_600_ (EC_50__OD600_) where promastigotes were persistently exposed to drug for a growth period of 72–96 h. Intracellular cAMP concentration was modulated by treatment with one hydrolyzable analog, 8-(4-chlorophenylthio)adenosine 3′,5′-cyclic monophosphate (8-pCPTcAMP) and one nonhydrolyzable analog, 8-(4-chlorophenylthio)adenosine-3′,5′-cyclic monophosphorothioate, Sp-isomer (Sp-8-pCPTcAMPS). Both the analogs significantly affected growth with (EC_50__OD600_) of 218.22 and 189.78 µM respectively (**Table 1**). On the flip-side, nonhydrolyzable cGMP-analog, 8-(4-chlorophenylthio)-guanosine 3′,5′-cyclic monophosphate (8-pCPT-cGMP) failed to show any antiproliferative effect on the parasites. Inhibitors previously determined to inhibit *Leishmania*-PDEs including EHNA, etazolate, dipyridamole, zaprinast, rolipram, and trequinsin were profiled for prospective antiproliferative activity. Dose–response curves were obtained by calculating the percentage proliferation against the untreated population after 4 days of growth to determine (EC_50__OD600_). Etazolate was found to be potently antiproliferative with (EC_50__OD600_) of 24.1 µM (**Table 1**). Dipyridamole and rolipram showed antiproliferative effects to lesser extents (EC_50__OD600_) of 61.3 and 29.04 µM respectively). Trequinsin, on the other hand, showed comparatively lower antiproliferative effect with (EC_50__OD600_) of 79.6 µM. The rest of the PDE inhibitors tested showed little effect on cell proliferation (**Table 1**). Interestingly, for rolipram and etazolate, marked differences were noted between EC_50__MTT_ and EC_50__OD600_, an observation that warrants further exploration on the dynamics of cytotoxicity induced by the inhibitors.

**Table 1:** Effect of cAMP analogs and PDE inhibitors on *L. donovani* promastigotes.

cAMP-analogs/PDE-inhibitors	(EC_50__OD600_) (µM)	(EC_50__MTT_) (µM)
8-pCPTcAMP	218.22 ± 37.24	200.34 ± 47.9
Sp-8-pCPTcAMPS	189.78 ± 29.01	250.23 ± 34.4
Etazolate	24.68 ± 6.31	89.23 ± 12.9
Dipyridamole	61.30 ± 11.71	112.33 ± 8.45
Trequinsin	79.59 ± 6.11	101.5 ± 15.56
Rolipram	29.04 ± 4.22	123.12 ± 20.05
EHNA	100.63 ± 14.63	156.77 ± 14.60
Zaprinast	265.15 ± 15.54	147.80 ± 14.9

EC_50__OD600_) was calculated after allowing the promastigotes to grow in the presence of analogs/inhibitors for 96 h. (EC_50__MTT_) was tested by MTT based vital assay after exposing the promastigotes to the analogs and inhibitors for 24 h. Data are representative of at least three independent experiments.

Since cAMP analogs and PDE inhibitors showed antiproliferative effects on the parasite, we were interested to introspect whether these impart any effect on parasite viability by determining (EC_50__MTT_) by MTT assay after 24 h of exposure. 8-pCPTcAMP and 8-Sp-pCPTcAMPS significantly decreased the parasite viability with (EC_50__MTT_) 200.34 and 250.23 µM respectively (**Figures S1A, B**; **Table 1**). 8-pCPTcGMP apparently did not affect parasite viability (data not shown). (EC_50__MTT_)values were also determined from the dose responsive curves of various PDE inhibitors (**Figure S1**). Etazolate, with an (EC_50__MTT_) of 89.23 µM, appeared as the most promising anti-proliferative of the inhibitors tested. Dipyridamole, trequinsin and rolipram showed EC_50_ values of 112.33, 101.5 and 123.12 µM respectively (**Table 1**, **Figures S1C–F**). All the other PDE inhibitors showed higher (EC_50__MTT_) values (**Table 1**). Collectively, these results suggest that cAMP analogs and PDE inhibitors affect parasite viability at higher doses.

### Effect of cAMP Analogs and PDE Inhibitors on the Cell Cycle

**Figure 1:**
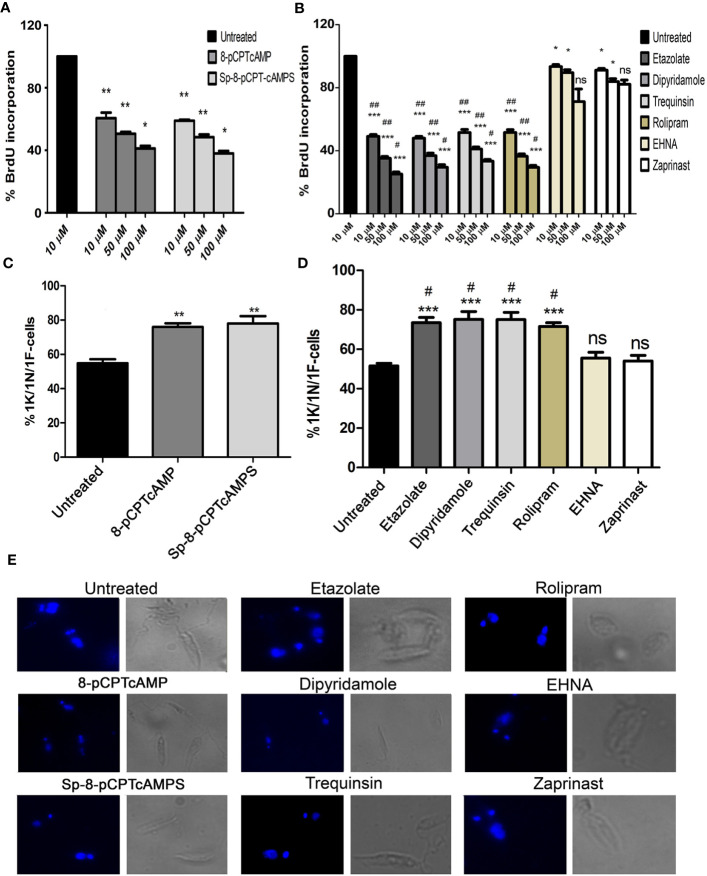
Effect of cAMP analogs and PDE inhibitors on the cell cycle. **(A**, **B)** Log phase promastigotes were pretreated with various concentrations of cAMP analogs **(A)** and PDE inhibitors **(B)** for 6 h followed by BrdU incorporation assay. **(C**, **D)** Log phase promastigotes were treated with cAMP analogs (100 μM) **(C)** and PDE inhibitors (50 μM) **(D)** for 12 h followed by DAPI staining, and 1K/1N/1F cells were scored for each of the treatment from at least 50 fields. Representative confocal microscopic images of the above experiment and Phase-contrast images of each treatment are shown separately **(E)**. Data are representative of three independent experiments. ***P < 0.001; **P < 0.01; *P < 0.05; ns, not significant compared to control untreated cells: ^##^P < 0.01; ^#^P < 0.05 compared to EHNA treated cells; two tailed unpaired t-test.

To assess the effect of cAMP analogs and PDE inhibitors on the cell cycle, promastigotes were treated with increasing concentrations (10, 50 and 100 µM) for 12 h followed by 2 h incubation with BrdU, and the percentage BrdU incorporation was studied by BrdU cell proliferation assay. 8-pCPTcAMP and 8-Sp-pCPTcAMPS treatment at 100 µM concentration significantly decreased the number of BrdU-positive cells (34.2 and 31.64% respectively compared to 82.3% in control) (**Figure 1A**), indicating fewer cells progressing into the S-phase of the cell cycle. Treatment with PDE inhibitors etazolate, dipyridamole, trequinsin, and rolipram also decreased the BrdU uptake significantly at 50 µM (24.13, 28.66, 31.86, and 26.56% compared to the untreated control of 82.38%), whereas PDE inhibitors EHNA and zaprinast showed only 73.76 and 68.5% positives at 50 µM respectively (**Figure 1B**). To further introspect the impact on cellular proliferation, progression of cell division was determined by scoring of the number, dimension, and position of the nucleus (N), kinetoplast (K) and flagellum (F) in cAMP analogs and PDE inhibitor-treated cells after labeling with DAPI. Cells with one of each of these organelles—1 nucleus/1 kinetoplast/1 flagella (1N/1K/1F) representing the G0/G1 population enter the S phase and duplicate their DNA content (1N^*^/1K^*^/1F cells, where * denotes duplicated DNA). Initiation of the growth of the second flagellum then begins (1N^*^/1K^*^/2F cells), where upon segregation of the two daughter kinetoplasts is initiated and 1N2K2F represents S phase followed by nuclear segregation (2N/2K/2F cells) which represents the G2/M phase population (Wheeler et al., 2011). Treatment with 8-pCPTcAMP and 8-Sp-pCPTcAMPS resulted in a marked increase in the 1N/1K/1F population from 54.8 to 75.93 and 78.03% respectively after 12 h, which was associated with concomitant decrease in the 2N/2K/2F and 1N/2K/2F populations (**Figures 1C, E**). Exposure to PDE inhibitors etazolate, rolipram, dipyridamole, and trequinsin resulted in a significant increase in the 1N/1K/1F cells (73.43 ± 4.3%, 75.16 ± 5.1%, 75 ± 4.9%, and 71.5 ± 6.4% compared to 51.53 ± 4.7% for control after 12 h) with equivalent decrease in 2N/2K/1F cells and 2N/2K/2F cells (**Figures 1D, E**). Treatment with PDE inhibitors zaprinast and EHNA (**Figure 1D**) showed N/K/F profiles comparable to that of the untreated cells. Taken together, these results suggest a probable G1 arrest of *Leishmania* promastigotes when they were treated with cAMP analogs and PDE inhibitors.

**Figure 2:**
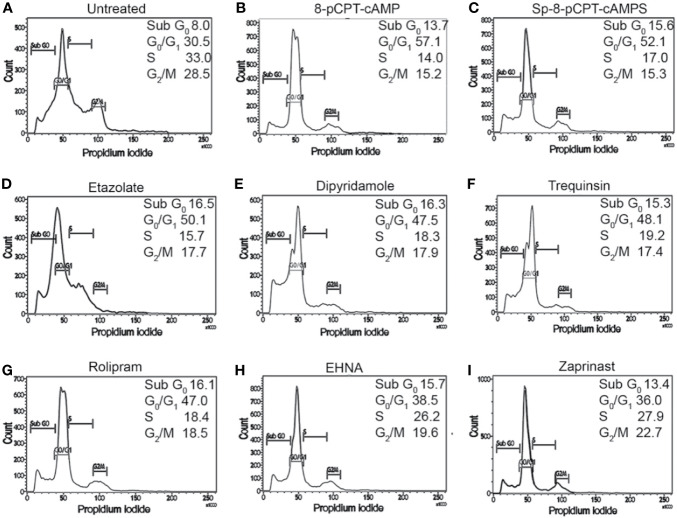
Impact of cAMP analogs and PDE inhibitors on cell cycle progression of *L. donovani* promastigotes. G1-synchronized log phase promastigote populations, either untreated **(A)** or treated with 8-pCPTcAMP (100 μM) **(B)**, Sp-8-pCPTcAMPS (100 μM) **(C)**; etazolate **(D)**, dipyridamole **(E)**, trequinsin **(F)**, rolipram **(G)**, EHNA **(H)** or zaprinast **(I)** (50 μM each) for 8, 16, and 24 h, were analyzed for cell cycle progression by propidium iodide staining based FACS analysis (**Tables S1**–**S3**). Representative histograms from 24 h data are presented.

To confirm the G_1_ phase cell cycle arrest, G_1_-phase-synchronized promastigotes were introduced into fresh medium, and the cell cycle profiles of cAMP analog-treated and PDE inhibitor-treated populations were studied for cellular DNA estimation by propidium iodide based FACS analysis. After exposing the G_1_ synchronized cells to 8-pCPTcAMP and Sp-8-pCPT-cAMPS (10, 50, and 100 µM), parasites were tracked over a period of 8, 16, and 24 h for cell cycle progression in the G_1_, S, and G_2_/M phase (**Figure 2A**, **Table S2** and **Table S3**). Treatment of the cells with 8-pCPT-cAMP reduced the S phase fraction indicating a potent role of 8-pCPTcAMP induced cell cycle arrest at the G_1_ phase, which also indicated by an increase in G1 population (**Figure 2B**, **Table S2** and **Table S3**). Similarly, Sp-8-pCPT-cAMPS reduced the percentages of S phase cells significantly and increased G1 phase population (**Figure 2C**, **Table S2** and **Table S3**). For treatment with PDE inhibitors etazolate, dipyridamole, and trequinsin, inhibitory toward *Leishmania* PDEA and PDEB (Johner et al., 2006), and rolipram, inhibitory toward *Leishmania* PDED (Biswas et al., 2017), showed significant G_1_ arrest after 24 h, while for inhibitors like EHNA and cGMP-PDE specific inhibitor zaprinst, no apparent G1 arrest was observed. Etazolate, dipyridamole, rolipram, and trequinsin resulted in significantly reduced S phase cell population at 100 µM respectively compared to the untreated population (**Table S2**, **Figures 2D–I** and **Table S3**). These results indicate that chemical modulation of intracellular cAMP can affect cell cycle progression.

### Modulation of Parasite Motility and ATP Level by Dose-Dependent Treatment of cAMP Analogs and PDE Inhibitors Is Linked to Mitochondrial Activity

**Figure 3:**
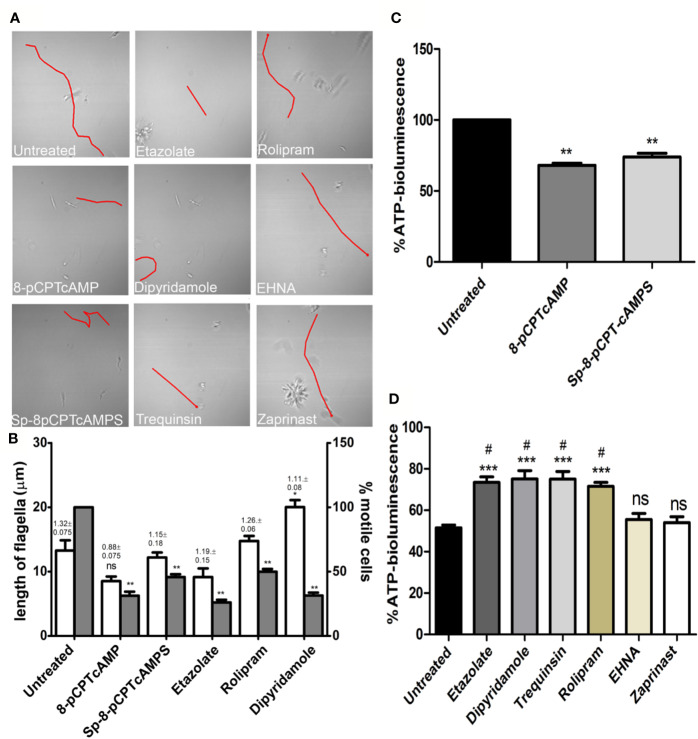
Inhibition of parasite motility and depletion of ATP-level upon administration of specific cAMP-analogs and PDE inhibitors. *Leishmania* promastigotes were treated with various cAMP-analogs (100 μM) and PDE inhibitors (50 μM), and the flagellar motility patterns of both the untreated and treated parasites were studied under the bright field of confocal microscope **(A)**. From the same set of experiments, flagellar length and ratio of flagellar length and cell body length were calculated (shown above each open bar). Percentage of motile cells was also calculated from the same set of experiment, represented by gray bar **(B)**. Levels of cellular ATP generation were studied after treating the parasites with cAMP-analogs **(C)** and PDE inhibitors **(D)** by ATP bioluminescence assay. Results are representative of three individual experiments, and the error bars represent mean ± SEM. **P < 0.01; *P < 0.05; compared to control untreated cells: ^#^P < 0.05 compared to EHNA treated cells; two tailed unpaired t-test. ns, not significant.

Since cAMP has a role in flagellar motility of *Leishmania* and social motility of *Trypanosoma* (Hill, 2010), an examination of their ﬂagellar motility patterns revealed that at a concentration of 100 µM both 8-pCPTcAMP and Sp-8-pCPTcAMPS affected the coordinated progressive flagellar movement or forward swimming motility of the parasites significantly (**Figure 3A**). Parasites were also significantly impaired for progressive flagellar movement in the presence of etazolate, dipyridamole, and rolipram (**Figure 3A**). On the other hand, EHNA and zaprinast did not affect forward flagellar movement (**Figure 3A**). Flagellar length and ratio of cell body–flagellar length have been associated with flagellar motility (Beneke et al., 2019). The average flagellar and cell body lengths were measured from 50 cells each from three independent populations of cells treated with the inhibitors. Apart from a minor increase for dipyridamole treatment, none of the cAMP analogs and PDE inhibitors inducted alteration in flagellar length. Parallel to such morphotypic alteration, the fraction of motile cells was significantly inhibited (>2-fold) upon the administration of cAMP analogs and also upon treatment with etazolate, dipyridamole, and rolipram (**Figure 3B**). Since one of the factors effecting flagellar motility is intracellular ATP generation, we determined the cellular ATP levels by ATP bioluminescent assay after treating the parasites with cAMP analogs and PDE inhibitors. Treatment of parasites with various concentrations of cAMP analogs and PDE inhibitors showed that at higher doses of cell permeable cAMP analogs and PDE inhibitors etazolate, dipyridamole, trequinsin, and rolipram significantly reduced the ATP levels (64.8 ± 5.8%, 61.0 ± 5.5%, 49.7 ± 4.1% and 54.7 ± 5.1% respectively) at a concentration of 50 µM, whereas 8-pCPTcAMP and Sp-8-pCPTcAMPS caused lesser effect (31.9 ± 2.8% and 26.0 ± 2.2% of reduction respectively) at 100 µM concentration (**Figures 3C, D**). Taking together these results suggest that higher concentrations of 8-pCPTcAMP, Sp-8-pCPTcAMPS, rolipram, dipyridamole, and etazolate significantly restrict flagellar movement and parasite motility with a concomitant decrease of cellular ATP levels. The PDE inhibitors like etazolate and rolipram are specific PDE4 inhibitors. Dipyridamole inhibits not only PDE4 but also other mammalian PDEs, IC_50_ values: 2μM for cGMP-activated PDE2, 3.3 μM for PDE4, and 0.8 μM towards PDE5 (Tetsi et al., 2017).

**Figure 4:**
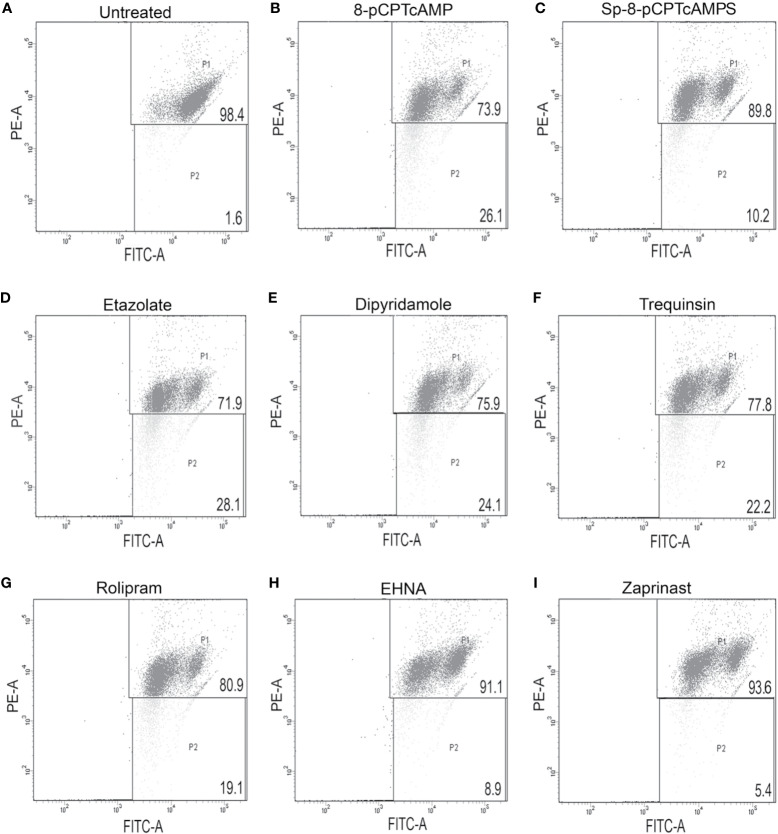
Modulation of mitochondrial membrane potential by cAMP analogs and PDE-inhibitors. Log phase promastigotes were incubated without **(A)** or with 8-pCPTcAMP (100 μM) **(B)**, Sp-8-pCPTcAMPS (100 μM) **(C)**, etazolate **(D)**, dipyridamole **(E)**, trequinsin **(F)**, rolipram **(G)**, EHNA **(H)** or zaprinast **(I)** (50 μM each) for 12 h. Mitochondrial membrane potential in the PDE inhibitor treated parasites was analyzed by FACS after treating them with JC-1, a vital dye used for estimating mitochondrial membrane potential. Representative scattered plots for three independent experiments are shown.

Since the ATP levels of cAMP analog-treated and PDE inhibitor-treated parasites were depleted, the impact of these on mitochondrial function was further elucidated by scoring mitochondrial membrane using JC-1. 8-pCPTcAMP induced maximum reduction in the mitochondrial membrane potential compared to control cells’ reduction (98.2 ± 1.1% for untreated *vs.* 74.6 ± 3.9 % for treated cells), whereas Sp-8-pCPTcAMPS induced moderate effect (98.2 ± 1.1% for untreated *vs.* 88.3 ± 5.1% for treated cells) at 100 µM (**Figures 4A–C**). Among the PDE inhibitors, etazolate and dipyridamole showed maximum reduction (98.2 ± 1.1% for untreated *vs.* 71.1 ± 5.3% and 75.2 ± 3.8% respectively for etazolate and dipyridamole treatment), whereas trequinsin and rolipram also showed significant effect (80.1 ± 5.8% and 76.1 ± 4.7% for rolipram and trequinsin treatment *vs.* 98.2 ± 1.1 % for the untreated). EHNA and zaprinast did not show significant effect (**Figures 4D–I**) compared to untreated control cells (**Table S4**).

### Etazolate Mitigates Intra-Macrophage Survival of Parasites

**Figure 5:**
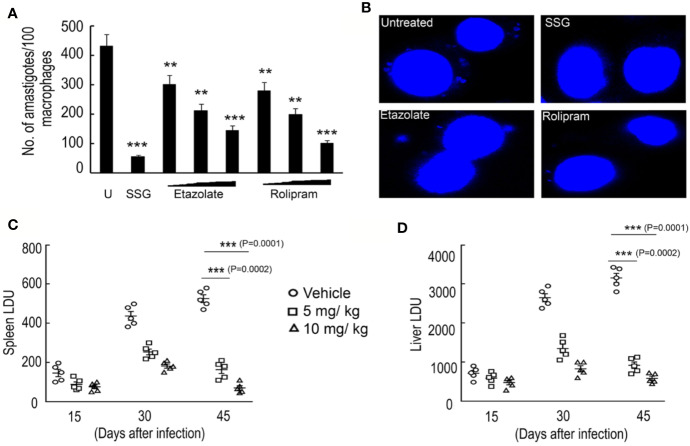
Effect of etazolate on parasite survival. Macrophages were preincubated with either increasing concentrations of rolipram and etazolate (1, 10, and 20 μM) or sodium stibogluconate (100 µg/ml) or left untreated for 30 min, followed by infection with *L. donovani* for another 72 h. The number of parasites per macrophage was scored for untreated (U) and treated systems by DAPI staining **(A)**. Representative microscopic images of infected macrophages either untreated or treated with sodium stibogluconate (100 µg/ml), rolipram (20 µM) or etazolate (20 µM) are shown **(B)**. BALB/c mice were infected with 10^7^ *L. donovani* promastigotes and treated etazolate (0–10 mg/kg/day). Etazolate was administered *i.p.* twice a week for 6 weeks starting at 1 week post-infection. PBS was used as vehicle control. The parasite burden in the liver and spleen was determined 6 weeks after infection and expressed as Leishman–Donovan Units (LDU). Disease progression was determined in the spleen **(C)** and liver **(D)** of mice that received etazolate at either 5 or 10 mg/kg/day twice weekly and parasite burden expressed as LDU at 2, 4, and 6 week post-infection **(C**, **D)**. Results are representative of three individual experiments, and the data represent mean ± SD. ***P < 0.001, **P < 0.01 unpaired two tailed t test.

Since etazolate showed maximum effect on cell cycle progression and caused mitochondrial membrane depolarization, the inhibitor was further explored as antileishmanial by analyzing its impact on parasite multiplication within RAW 264.7 macrophages. Etazolate apparently had no effect on viability of macrophage as determined by MTT assay and gross cell morphology analysis as visualized by microscopy up to 200 µM (data not shown). Etazolate treatment resulted in significant reduction in the number of parasites within macrophages with a maximum effect (75.0 ± 6.7% reduction) observed at 100 µM concentration (**Figures 5A, B**).

Rolipram, though was not as efficient as etazolate in modulating cell cycle progression or mitochondrial membrane potential of the parasite, showed significant decrease in intra macrophage infectivity. But, considering the scientific facts which stress upon the adverse effects of Rolipram in drug trials (Zhu et al., 2001; Kumar et al., 2013), etazolate was tested in BALB/c mice to test its efficacy to clear parasitic burden from the spleen and liver in infected groups. A dose of 5 and 10 mg/kg/day was administered orally over a period of 10 days following 1 month of *Leishmania* infection in mice. Spleen and liver were removed from treated and untreated 45 day infected mice, and multiple impression smears were prepared and stained with Giemsa. Spleen or liver parasite burdens, expressed as Leishman–Donovan units (LDU), were calculated as the number of parasites per 1,000 nucleated cells/organ weight (in grams). A dose-related inhibition was noted at a dose of 5 and 10 mg/kg/day with great reduction of liver and spleen parasite burden by 70.7 ± 6.9% and 79.5 ± 7.2% compared to untreated infected mice (**Figures 5C, D**). These results highlight etazolate as candidate antileishmanial.

## Discussion

Leishmaniasis represents a significant global burden and poses a great challenge to drug discovery and delivery because of the intracellular nature and disseminated locations of the parasite. Most of the current modes of treatment available till date are not only toxic but also do not completely cure, *i.e.*, eliminate the parasite from infected individuals (Wheeler et al., 2011). The problem of increased chemoresistance towards the current set of available drugs is further complicating the treatment for this disease (Amato et al., 2008; Beneke et al., 2019). Because treatment is a growing problem, the development of new medicines that can replace or complement the current set of available therapeutic compounds is necessary.

cAMP has been emerging as one of the major modulator of cytological events in kinetoplastida parasites (Tagoe et al., 2015). Despite the identification of several cyclic nucleotide binding effector molecules, there is a severe deficit of comprehensive knowledge on cyclic nucleotide signaling from receptor to effector till date. The candidate regulatory subunits and cyclic nucleotide binding domains bind cAMP/cGMP with extremely high and beyond physiologically permissible K_d_ indicating almost the absence of effective interaction and to perform as a typical effector under physiological condition (Wang et al., 2007) is not feasible. Bachmaier et al. (Bachmaier et al., 2019) recently reported binding of nucleotides such as 7-deazapurine derivatives to PKA-regulatory subunit of *T. brucei* instead of the canonical cAMP or cGMP. While studying the resistance against PDE inhibitor cpdA and cpdB, a novel class of cAMP effectors, CARP was identified in *T. brucei* (Gould et al., 2013), indicating the existence of noncanonical cAMP effectors in kinetoplastida parasites. CARP and its orthologs (Gould et al., 2013) are conserved among several kineoplastida parasites. On the receptor/cAMP generator side of the pathway, an oxygen stimulated soluble adenylyl cyclase has been identified; however since this gene is absent in *Trypanosoma*, this enzyme probably contributes to some cAMP-dependent function restricted to *Leishmania*. The activation of conserved receptor adenylyl cyclases possibly serves as the major trigger for cAMP, though the “ligands” or specific conditions for triggering such “receptors” are not defined yet. The phenotypic attributes like cell death, mitochondrial depolarization, and G1 arrest illustrated in this study or social motility (Ouellette et al., 2004), flagellar wave reversal (Mukhopadhyay and Dey, 2016) are expected to be linked with it or some unidentified upstream effectors. Interestingly 8-pCPTcAMP is less antiproliferative to *L. donovani* compared to *T. brucei* (Gould et al., 2013; Sen Santara et al., 2013), indicating variation of response against this cAMP-analog between trypanosomatids. Moreover, the nonhydrolyzable analog Sp-8-pCPTcAMPS elicited comparable response in terms of cell cycle analysis and mitochondrial functioning. Such observations hint that possibly the antiproliferative/differentiation inducing action of hydrolysis product 8-pCPTcAMP as observed in *T. brucei* (Laxman et al., 2006) is not conserved in *Leishmania*. However, the EC_50_ values for the cAMP-analogs, determined in this study, showed subtle deviation across experiments, suggesting responsiveness against the analogs is delicately linked to the physiological state of the tested *L. donovani* population. Intensive forward genetic approach combined with systemic analysis might shed light on the mechanistic details of cAMP mediated responses and variations among trypanosomatids.

Though cAMP has a cytoprotective effect against oxidative stress in *Leishmania*, in *Trypanosma* cAMP-phosphodiesterase inhibitor cpdA displays potent antitrypanosomal activity (Gould et al., 2013), indicating that long term potentiation of cAMP triggering probably induces cell death in kinetoplastida parasites. Such observations provide genetic and pharmacological validation of using PDEs as novel drug targets for diseases caused by the kinetoplastid parasites including *Leishmania* (Sebastián-Pérez et al., 2018). Sebastián-Pérez et al. already projected PDE-inhibitory imidazole derivatives as candidate antileishmanial; our study corroborates PDEs as promising targets and explores the possibility of repurposing human PDE inhibitors as the starting framework for the design of evaluating the use of inhibitors with substantial data for human application. This proposition is supported by the inhibitory effects of some human PDE inhibitors observed on *T. cruzi* PDEC; *e.g.* etazolate inhibits human PDE4 and TcrPDEC with IC_50_ values of 2 and 0.7 μM (Wang et al., 2012). Among the PDE inhibitors used in this study, etazolate, a pyrazolopyridine derivative, showed maximum antiproliferative activity against *Leishmania* parasites with least cytotoxic effect on macrophage cells cultured *in vitro*. Further, it was observed that it significantly affected the cell cycle progression and mitochondrial membrane potential of the parasite, therefore we assessed *in vitro* their ability to clear parasite load within the macrophage cells. Though initial reports in *T. brucei* suggested that etazolate inhibits growth substantially though it does not affect intracellular cAMP pool in the parasite, a number of studies suggested that it can potentially inhibit specific PDEs from *T. cruzi* and at least two PDE from *Leishmania* (Wang et al., 2012; Vij et al., 2014). In this context, measuring the impact of etazolate in intracellular cAMP level would have suggested direct correlation between etazolate action and elevation of cAMP by PDE inhibition. Since etazolate apparently has no impact on total cellular cAMP pool in *T. brucei* (Richard and Werbovetz, 2010), it is anticipated that antiparasitic activity of etazolate is due to some unidentified effector(s) or microdomain-specific modulation of cAMP response. Such alterations of subcellular second messenger dynamics are often not reflected in the chromogenic ELISA based methods dealing with whole cell lysates with sensitivity around 20 nM. Development of a FRET based fluorescence resonance energy transfer (FRET)-based sensor to monitor cAMP dynamics at subcellular level (Oberholzer et al., 2015) for *Leishmania* will shed light on the precise mechanism of cytological events kindled by PDE inhibitors.

Among the first generation of well-known and clinically tested PDE4 inhibitors, rolipram was a prototype as it was the first selective PDE4 inhibitor to be studied with potent anti-inflammatory actions. Rolipram was also the first blood-brain-barrier (BBB) permeable PDE4 inhibitor, developed for ameliorating neuroinflammation which showed potent effectiveness in various animal models, including depression, neuropathic pain, Alzheimer’s disease, Parkinson’s disease, and multiple sclerosis (Rose et al., 2005; Garcia-Osta et al., 2012; Pearse and Hughes, 2016). However, the clinical application of rolipram is limited because of its behavioral and other severe side effects like nausea, vomiting, and severe adverse reactions in clinical trials (Zhu et al., 2001; Kumar et al., 2013). Hence, a second generation of more selective PDE4 inhibitors has been prepared in practice and is under various levels of investigation (Kumar et al., 2013). Etazolate, primarily a selective PDE4 inhibitor (Bachmaier et al., 2019; Shaw et al., 2019) on the other hand, has been shown as a well-established drug of choice with no major side effects reported (Shaw et al., 2019). Etazolate is currently in the third phase of clinical evaluation for Alzheimer’s disease (Vellas et al., 2011). Etazolate, in preclinical studies as well as pharmacokinetic and safety profiles in Phase I and Phase II clinical studies, shows promise. Etazolate produced antidepressant-like effects in animal models of depression, and at the same time it could be used in the treatment of Alzheimer’s disease. Moreover, chronic etazolate treatment at a dose between 0.5 and 1 mg/kg treatment significantly diminished traumatic brain injury induced anomalies (Wang et al., 1997). Earlier the drug was demonstrated to be well-tolerated and devoid of side effects by Marcade et al. (2008) based on their study on guinea pigs where they administered the drug at a dose of 10 mg/kg for 15 days (Marcade et al., 2008; Drott et al., 2010). In coherence to these reports and based on our *in vitro* cytotoxicity analysis on RAW264.7 cell line in mice, we tested a dose range between 5 and 20 mg/kg which appeared to be well tolerated by the mice. With its potential inhibitory impact on amastigote proliferation *in vitro* and in experimental animal models for VL, etazolate can be considered a drug for repurposing against VL and can serve as a candidate molecule for synthesizing more selective drugs. Bearing in mind the urgent need for new therapies and the desirability of a relatively short duration of treatment, the requirement for very stringent selectivity might be relaxed somewhat if the drug was efficacious, and its side effects were acceptable and reduced compared to current available treatments. Devoid of gross side effects, etazolate, as suggested by the observations, can be exploited for developing intervention strategies against leishmaniasis.

## Data Availability Statement

All datasets generated for this study are included in the article/**Supplementary Material**.

## Ethics Statement

The animal study was reviewed and approved by Animal Ethics Committee on Animal Experiments of the University of Kalyani (892/GO/Re/S/01/CPCSEA).

## Author Contributions

Conceptualization: AruB, AriB, PD. Investigation: AS, AniB, AV, AruB. Methodology: AruB, AS, AriB. Project Administration: AruB, AriB, PD. Resources and Funding: AruB. Supervision: AruB, AriB. Validation: AS, AniB, AruB. Draft writing: AS, AniB, AV. Review and Editing (writing): AruB, AriB, PD.

## Funding

AruB received grant from DST-INSPIRE Faculty Programme (IFA-12 LSBM-22), Govt. of India.

## Conflict of Interest

The authors declare that the research was conducted in the absence of any commercial or financial relationships that could be construed as a potential conflict of interest.
