# Supplementary material for: Evaluation of Modulators of cAMP-Response in Terms of Their Impact on Cell Cycle and Mitochondrial Activity of Leishmania donovani
Source: Front Pharmacol. 2020 May 29;11:782. doi: 10.3389/fphar.2020.00782 (PMC7326082; doi:10.3389/fphar.2020.00782)
Supplement: Supplementary file 1 [file DataSheet_1.docx]

**Supplementary data:**

Figure S1. Dose responsive viability assay for cAMP analogue and PDE inhibitor

treatments.

Table- S1. Cell cycle analysis of *L. donovani*promastigotes in presence of cAMP analogues.

Table-S2. Cell cycle analysis of *L. donovani*promastigotes in presence of PDE inhibitors.

Table-S3. Cell cycle phase distribution of *L. donovani* promastigotes after 24 h treatment of cAMP-analogues and PDE inhibitors.

Table-S4. Modulation of mitochondrial membrane potential upon administration of cAMP analogues and PDE-inhibitors.


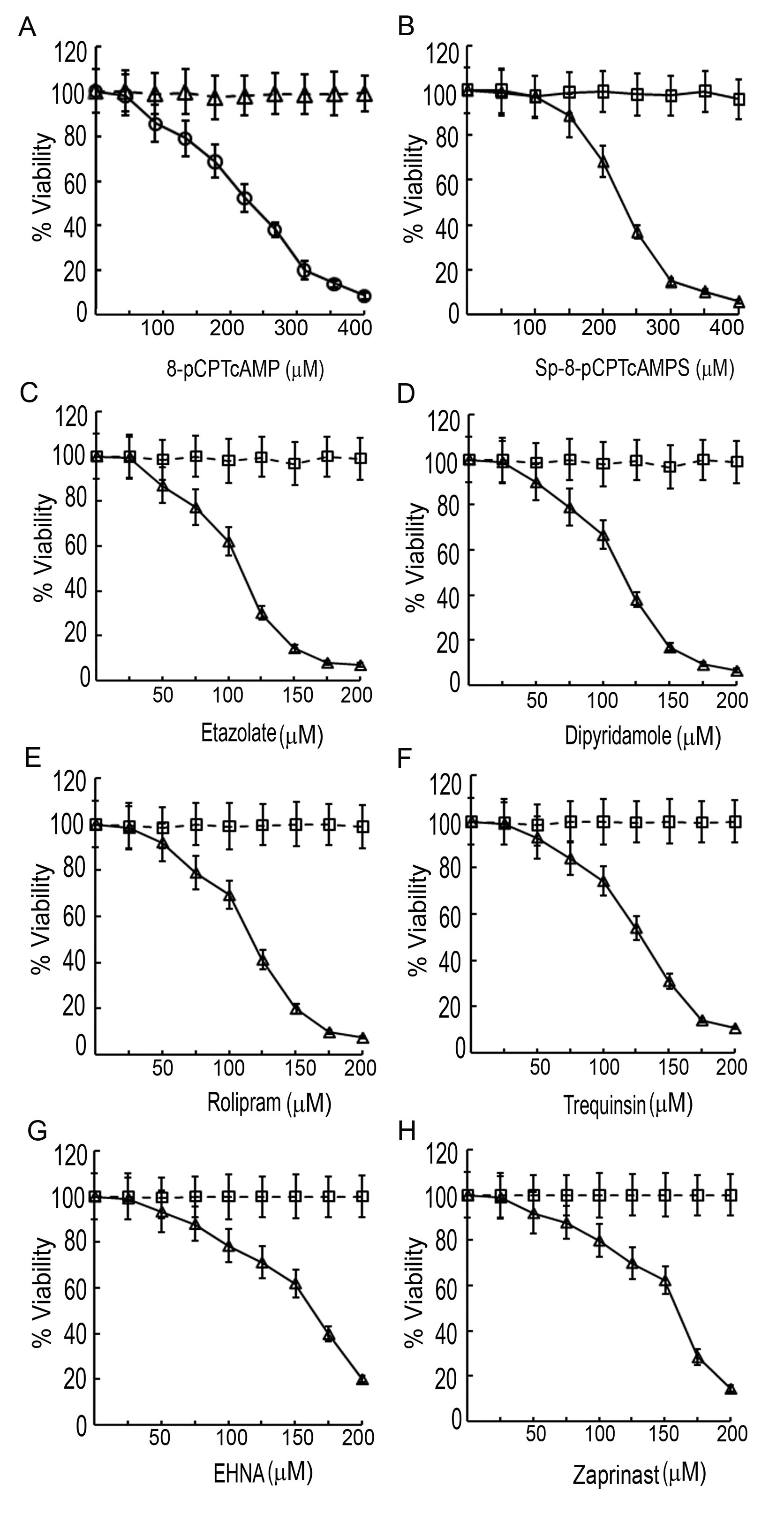


**Figure S1.** **Dose responsive viability assay for cAMP analogue and PDE inhibitor treatments.** Log phase promastigotes were pretreated with various concentrations of 8-pCPTcAMP (A) and Sp-8-pCPTcAMPS (B); etazolate (C), dipyridamole (D), trequinsin (E), rolipram (F), EHNA (G) or zaprinast (H) for 24h followed by MTT assay. Dotted lines represent untreated promastigotes analyzed in parallel to treated population.

Table-S1. **Cell cycle progression after cAMP-analogue treatment.** G1 synchronized *L. donovani* promastigotes were exposed to various doses of cAMP analogues. Cell cycle stage specific distribution of the populations were analyzed by propidium iodide nuclear staining based FACS analysis after various time periods.

| Treatment | Duration | % Sub G_0_ | % G_0_/G_1_ | % S | % G_2_/M |
| --- | --- | --- | --- | --- | --- |
| Control | 8 h | 16.4 | 39.4 | 26.2 | 18 |
|  | 16 h | 11.1 | 37.15 | 31.2 | 19.85 |
|  | 24 h | 6.5 | 35.3 | 32.6 | 25.6 |
| 8-pCPTcAMP (10 µM) | 8 h | 14.03 | 43.87 | 26.3 | 15.8 |
|  | 16 h | 18.19 | 46.81 | 18.2 | 16.8 |
|  | 24 h | 15.8 | 52.1 | 16 | 18.7 |
| 8-pCPTcAMP (50 µM) | 8 h | 12.3 | 48.8 | 24 | 14.9 |
|  | 16 h | 15.1 | 51.1 | 15.3 | 18.5 |
|  | 24 h | 16.1 | 56.1 | 14.2 | 13.6 |
| 8-pCPTcAMP (100 µM) | 8 h | 12.1 | 47.8 | 23.8 | 16.3 |
|  | 16 h | 14.6 | 53 | 14.1 | 18.3 |
|  | 24 h | 13.7 | 57.1 | 14 | 15.2 |
| Sp-8-pCPTcAMPS (10 µM) | 8 h | 13.6 | 40.1 | 25.1 | 21.6 |
|  | 16 h | 15 | 44.1 | 21.1 | 19.8 |
|  | 24 h | 15.8 | 47.1 | 18.4 | 18.7 |
| Sp-8-pCPTcAMPS (50 µM) | 8 h | 13.4 | 45.1 | 24.1 | 17.4 |
|  | 16 h | 14.1 | 47.7 | 20.2 | 18 |
|  | 24 h | 15.1 | 51.4 | 17.6 | 15.9 |
| Sp-8-pCPTcAMPS (100 µM) | 8 h | 14.1 | 44.9 | 23 | 18 |
|  | 16 h | 14.8 | 49.1 | 18.9 | 17.2 |
|  | 24 h | 15.6 | 52.1 | 17 | 15.3 |

Table-S2.**Cell cycle progression after PDE inhibitor treatment.**G1 synchronized *L. donovani* promastigotes were exposed to various doses of PDE inhibitors. Cell cycle stage specific distribution of the populations were analyzed by propidium iodide nuclear staining based FACS analysis after various time periods.

| Treatment | Duration | % Sub G_0_ | % G_0_/G_1_ | % S | % G_2_/M |
| --- | --- | --- | --- | --- | --- |
| Control | 8 h | 16 | 40 | 25 | 19 |
|  | 16 h | 10.5 | 34.5 | 29.5 | 23.5 |
|  | 24 h | 8 | 30.5 | 33 | 28.5 |
| Etazolate (50 µM) | 8 h | 16.5 | 41.2 | 23.5 | 18.8 |
|  | 16 h | 16.9 | 43.8 | 20.4 | 18.9 |
|  | 24 h | 17 | 45.1 | 17.8 | 20.1 |
| Etazolate (100 µM) | 8 h | 15.7 | 42 | 23.2 | 19.1 |
|  | 16 h | 16 | 44.8 | 18.7 | 20.5 |
|  | 24 h | 16.5 | 50.1 | 15.7 | 17.7 |
| Dipyridamole (50 µM) | 8 h | 16.2 | 41.2 | 24.8 | 17.8 |
|  | 16 h | 14.7 | 48.1 | 20.1 | 17.1 |
|  | 24 h | 15 | 49 | 19.1 | 16.9 |
| Dipyridamole (100 µM) | 8 h | 14.5 | 41.8 | 24 | 19.7 |
|  | 16 h | 14.9 | 46.1 | 19.1 | 19.9 |
|  | 24 h | 16.3 | 47.5 | 18.3 | 17.9 |
| Rolipram (50 µM) | 8 h | 16 | 39.3 | 25.5 | 19.2 |
|  | 16 h | 15.4 | 41.7 | 23.1 | 19.8 |
|  | 24 h | 15.8 | 44.1 | 21.5 | 18.6 |
| Rolipram (100 µM) | 8 h | 15.7 | 40.8 | 23 | 20.5 |
|  | 16 h | 16 | 45.6 | 20.8 | 17.6 |
|  | 24 h | 16.1 | 47 | 18.4 | 18.5 |
| Trequinsin (50 µM) | 8 h | 14.8 | 40.1 | 25.2 | 19.9 |
|  | 16 h | 15.6 | 45.1 | 22.5 | 16.8 |
|  | 24 h | 16 | 48 | 20.1 | 15.9 |
| Trequinsin (100 µM) | 8 h | 16 | 44 | 24.1 | 15.9 |
|  | 16 h | 15.1 | 47 | 20.1 | 17.8 |
|  | 24 h | 15.3 | 48.1 | 19.2 | 17.4 |
| EHNA (50 µM) | 8 h | 15.1 | 40.1 | 25.4 | 19.4 |
|  | 16 h | 13.4 | 36.7 | 27.8 | 22.1 |
|  | 24 h | 14 | 34.22 | 29.1 | 22.68 |
| EHNA (100 µM) | 8 h | 15.4 | 39.8 | 24.6 | 20.2 |
|  | 16 h | 16.7 | 39.1 | 25 | 19.2 |
|  | 24 h | 15.7 | 38.5 | 26.2 | 19.6 |
| Zaprinast (50 µM) | 8 h | 15.9 | 40 | 24 | 20.1 |
|  | 16 h | 14.9 | 37.3 | 26.7 | 21.1 |
|  | 24 h | 14.1 | 34.8 | 28 | 23.1 |
| Zaprinast (100 µM) | 8 h | 14.5 | 39.4 | 25 | 21.1 |
|  | 16 h | 13 | 38.1 | 27 | 21.9 |
|  | 24 h | 13.4 | 36 | 27.9 | 22.7 |

Table-S3. **Cell cycle phase distribution of *L. donovani* promastigotes after 24 h treatment of cAMP-analogues and PDE inhibitors.** Cell cycle stage specific distribution of the populations was analyzed by propidium iodide nuclear staining based FACS analysis after 24h.Results are representative of three individual experiments, and the data represent mean ± SD. **P<0.01, *P<0.05, ns not significant compared to control untreated cells: ^#^P<0.05 compared to EHNA treated cells; unpaired two tailed t test.

| Treatment | Duration | % Sub G_0_ | % G_0_/G_1_ | % S | %G2/M |
| --- | --- | --- | --- | --- | --- |
| Control | 24h | 8.1±1.6 | 31.7±3.0 | 33.5±2.6 | 27.3±2.5 |
| 8-pCPTcAMP (100 µM) | 24h | 13.2±1.9 | 57.9±4.7^*/#^ | 13.6±2.8 | 15.5±2.1 |
| Sp-8-pCPTcAMPS (100 µM) | 24h | 16±2.3 | 52.9±4.5^**/#^ | 16.7±1.7 | 16.2±1.6 |
| Etazolate (100 µM) | 24h | 16.9±1.9 | 51.2±3.1^**/#^ | 15.1±1.7 | 17.2±1.8 |
| Dipyridamole (100 µM) | 24h | 16±1.3 | 47.4±2.8^**/#^ | 17±1.2 | 18.1±1.5 |
| Rolipram (100 µM) | 24h | 16.8±1.7 | 47.5±3.7^**/#^ | 18.2±2.6 | 18±1.6 |
| Trequinsin (100 µM) | 24h | 14.8±1.5 | 46.4±3^**/#^ | 18.7±2.4 | 17.8±2.8 |
| EHNA (100 µM) | 24h | 15.3±1 | 36.9±4.5^ns^ | 26.5±1.8 | 19.3±2.5 |
| Zaprinast (100 µM) | 24h | 14.1±2 | 37.1±2.7^ns^ | 27.5±2.8 | 24.1±1.4 |

Table-S4. **Modulation of mitochondrial membrane potential upon administration of cAMP analogues and PDE-inhibitors.** Mitochondrial membrane potential in the parasites were analysed by FACS after treating them with using JC-1, a mitochondrial vital dye. Results of % cells above cut-off are representative of three individual experiments, and the data represent mean ± SD. ***P<0.001, *P<0.05, ns not significant compared to control untreated cells. ^##^P<0.01, ^#^P<0.05 compared to EHNA treated cells; unpaired two tailed t test.

| Treatment | % of cells |
| --- | --- |
| Control | 98.2±1.1 |
| 8-pCPTcAMP (100 µM) | 74.6±3.9^***/##^ |
| Sp-8-pCPTcAMPS (100 µM) | 88.3±5.1^*/#^ |
| Etazolate (100 µM) | 71.1±5.3^***/##^ |
| Dipyridamole (100 µM) | 75.2±3.8^***/##^ |
| Rolipram (100 µM) | 80.1±5.8^***/#^ |
| Trequinsin (100 µM) | 76.1±4.7^***/#^ |
| EHNA (100 µM) | 92.1±4.2^ns^ |
| Zaprinast (100 µM) | 93.2±3.9^ns^ |
